# Anemia: An unusual cause of free-floating thrombus of carotid artery

**Published:** 2019-04-04

**Authors:** Maziar Emamikhah, Narges Yazdi, Nafiseh Mohebi, Monireh Eslami, Mehdi Moghaddasi

**Affiliations:** 1 Department of Neurology, School of Medicine, Iran University of Medical Sciences, Tehran, Iran; 2 Rasool-e-Akram Hospital, Iran University of Medical Sciences, Tehran, Iran

**Keywords:** Carotid Thrombosis, Carotid Artery, Common, Doppler Ultrasound, Anemia, Thrombocytosis, Carotid Endarterectomy

Free-floating thrombus (FFT) of carotid artery is a rare condition of all carotid artery diseases leading to stroke. Less than 150 cases have been reported in the literature.^1^ Various etiologies have been presumed among which atherosclerosis is the most common. Non-atherosclerotic cases are even much scarcer to be observed in daily practice. Here, we present a patient with FFT suffering from anemia, with no atherosclerotic disease, managed successfully with a combined medical and surgical approach.

A 49-year-old man admitted to our tertiary care center, Rasoul-Akram hospital affiliated to Iran University of Medical Sciences, Tehran, Iran, for colostomy reversal surgery following abdominal stab wound one year earlier. The reversal surgery was, and after postanesthesia recovery, patient was unable to speak and move his right limbs. Physical examination revealed global aphasia and right hemiparesis. He was ex-smoker, but had no other risk factor for atherosclerosis or hypercoagulability state.

The initial brain magnetic resonance imaging (MRI) study showed multiple cortical hypersignality in left frontoparietal lobe with restriction in diffusion weighted imaging (DWI) and apparent diffusion coefficient (ADC) sequences, favoring acute infarction in the territory of left middle cerebral artery (MCA) (Figure 1).

**Figure 1:**
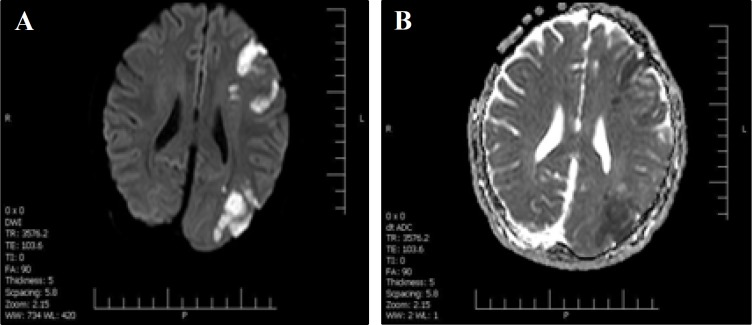
Diffusion weighted imaging (DWI) (A) and apparent diffusion coefficient (ADC) map (B) sequences of brain magnetic resonance imaging (MRI). Multiple cortical signal changes are evident in the left frontoparietal lobe with restriction in the DWI-ADC sequences, in favor of acute infarction in the territory of left middle cerebral artery (MCA)

Electrocardiogram (ECG) and transesophageal echocardiography (TEE) were entirely normal. Duplex ultrasonography of carotid arteries showed a large isoechoic intraluminal lesion compatible with fresh floating thrombosis in left common carotid artery (CCA), 6 cm proximal to bifurcation, along with turbulent and circumferential flow and increased peak systolic velocity (PSV) in CCA, and normal pattern and velocity in internal and external carotid arteries (Figure 2). The intima-media thickness (IMT) was within normal limits, and no atherosclerotic plaque was detected. Additionally, no microembolic signal (MES) was detected during 10 minutes transcranial Doppler (TCD). 

**Figure 2:**
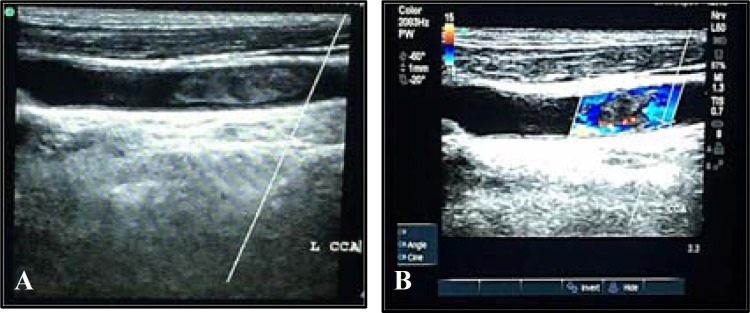
B-mode duplex ultrasonography of left common carotid artery showed an isoechoic intraluminal lesion compatible with fresh floating clot (A), turbulent, and circumferential blood flow in the intraluminal thrombosis (B).

Computerized tomography angiogram (CTA) confirmed the presence of FFT in left CCA. In axial and coronal planes, the so called “donut sign” and “finger sign” were evident, respectively (Figure 3).

The comprehensive laboratory investigation including vasculitis and hypercoagulation tests were entirely normal, except for anemia (lowest hemoglobin level was 9 g/dl) and thrombocytosis (highest platelet count was 600000 per µl) after surgery.

Following duplex ultrasonography findings, the patient underwent anticoagulation with unfractionated heparin (UFH) after which urgent left carotid endarterectomy (CEA) was performed and the thrombus was removed. Anticoagulation was continued for 10 days followed by dual antiplatelet drugs (aspirin and Plavix) and statin. The patient was discharged after partial symptoms amelioration. No embolic or hemorrhagic complication occurred neither during admission nor during three months of follow up.

FFT is defined as an elongated thrombus attached to the arterial wall with circumferential blood flow at its distal most aspect with cyclical motion relating to cardiac cycles. Internal carotid artery (ICA) stands for the most prevalent site of thrombosis, 75 percent of all cases. CCA and carotid bifurcation are the next (7%). A wide range of incidence rate, 0.05 to 1.45 percent, has been reported so far with a male predominance and younger age group.^1^ Several overlapping pathologies are implicated to be the cause of FFT including intraluminal thrombus, embolic thrombus, plaque thrombus, or mobile thrombus. It is said that atherosclerosis and hypercoagulate state are the leading etiologies of carotid thrombus formation.^1^ But, as mentioned sparsely in literature, other reported causes are hyperfibrinogenemia, iron deficiency anemia and thrombocytosis, stimulant drugs, carotid aneurysms, arterial dissections, malignancy, paradoxical embolization through a patent foramen ovale (PFO), and meningioma.^2^^-^^5^

**Figure 3:**
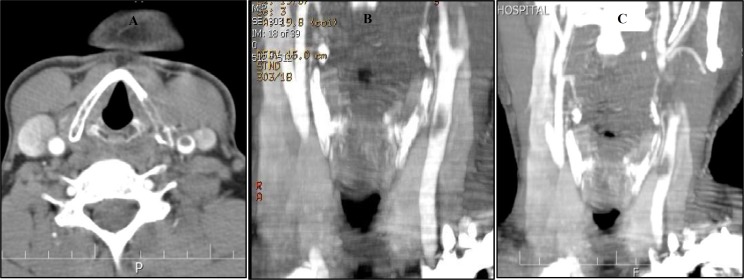
Computerized tomography angiogram (CTA) shows a free-floating thrombus (FFT) in left common carotid artery (CCA) attached to medial intima (donut sign) (A), mural attachment of clot (B), and mural attachment plus distal floating part of thrombus (finger sign) (C)

With regard to our patient, no common cause of carotid thrombus formation was explored but anemia accompanied with thrombocytosis. There are two case reports of carotid thrombosis associated with anemia,^6^^,^^7^ which introduced with twenty years interval, two young patients with acute stroke suffering from severe iron deficiency anemia secondary to menorrhagia, and probably reactive thrombocytosis. Diagnosis is made by duplex ultrasonography of carotid, as a fast noninvasive test, and can be confirmed by CTA.^1^^,^^2^ In this case, urgent duplex ultrasonography was performed as screening test, and then CTA to confirm, both of which seemed to be quite diagnostic. As an infrequent medical condition, there is no unified consensus for FFT management. Medical therapy alone, medical therapy with delayed surgery, and medical therapy with urgent CEA, all have been tried, and the latest seems to be safe and as mentioned by Ferrero, et al.^2^ may even be superior. 

Since free intramural thrombosis in carotid artery might be overlooked specially in otherwise healthy patients, this case presentation is to emphasize on prompt diagnosis, possible unusual etiologies, and effective treatment to prevent further devastating complications.

## Notes:

**How to cite this article:** Emamikhah M, Yazdi N, Mohebi N, Eslami M, Moghaddasi M. Anemia: An unusual cause of free-floating thrombus of carotid artery. Iran J Neurol 2019; 18(2): 87-9.
